# Predicting the Impact of Describing New Species on Phylogenetic Patterns

**DOI:** 10.1093/iob/obz028

**Published:** 2019-11-07

**Authors:** D C Blackburn, G Giribet, D E Soltis, E L Stanley

**Affiliations:** 1 Department of Natural History, Florida Museum of Natural History, University of Florida, Gainesville, FL 32611, USA; 2 Department of Organismic and Evolutionary Biology and Museum of Comparative Zoology, Harvard University, Cambridge, MA 02138, USA

## Abstract

Although our inventory of Earth’s biodiversity remains incomplete, we still require analyses using the Tree of Life to understand evolutionary and ecological patterns. Because incomplete sampling may bias our inferences, we must evaluate how future additions of newly discovered species might impact analyses performed today. We describe an approach that uses taxonomic history and phylogenetic trees to characterize the impact of past species discoveries on phylogenetic knowledge using patterns of branch-length variation, tree shape, and phylogenetic diversity. This provides a framework for assessing the relative completeness of taxonomic knowledge of lineages within a phylogeny. To demonstrate this approach, we use recent large phylogenies for amphibians, reptiles, flowering plants, and invertebrates. Well-known clades exhibit a decline in the mean and range of branch lengths that are added each year as new species are described. With increased taxonomic knowledge over time, deep lineages of well-known clades become known such that most recently described new species are added close to the tips of the tree, reflecting changing tree shape over the course of taxonomic history. The same analyses reveal other clades to be candidates for future discoveries that could dramatically impact our phylogenetic knowledge. Our work reveals that species are often added non-randomly to the phylogeny over multiyear time-scales in a predictable pattern of taxonomic maturation. Our results suggest that we can make informed predictions about how new species will be added across the phylogeny of a given clade, thus providing a framework for accommodating unsampled undescribed species in evolutionary analyses.

## Introduction

As we describe new species, we reshape our knowledge of evolution. Each new species is not only a name added to an ever-growing global inventory but also a new branch of the Tree of Life, representing novel natural history, ecology, physiology, and phenotypes. While it is difficult to make predictions about the biology of undiscovered species, we should be able to make an educated guess as to the effects of adding newly described species to a phylogeny.

We know that many species remain undescribed (Bickford et al. 2007; Pimm et al. 2010; Stork et al. 2015) and that incomplete taxon sampling affects our ability to infer phylogenetic relationships and macroevolutionary processes (Nee et al. 1994; Graybeal 1998; Pybus and Harvey 2000; Heath et al. 2008a, 2008b; Brock et al. 2011; Rabosky 2015). Further, discovery and description of new species can be biased by factors including geographic distribution, body size, and conspicuousness (Blackburn and Gaston 1995; Gaston et al. 1995; Alroy 2003; Collen et al. 2004). We expect that clades vary in the degree to which species diversity is documented due to differences, for example, in the ease of collection or the difficulty of diagnosing distinct species using morphological data. While analyses might accommodate incomplete sampling by comparing observed trees to simulated sets of trees with more species (e.g., Rabosky and Lovette 2008; Shi and Rabosky 2015), this approach can be undermined if unsampled species are not randomly distributed across a phylogeny. It is therefore important to understand how newly described species might be distributed across clades. Will these species add short branches near the tips of a phylogeny? Or, in contrast, will these species add a broader diversity of branch lengths including long branches to deep nodes? After all, the discovery of a new species of pocket mouse has a different impact on our understanding of mammalian evolution than the discovery of a species belonging to a new order of mammals. To address these questions, we need tools to evaluate the extent to which the phylogeny of a taxon is already known.

Using two sources of data—a phylogeny in which the tips are species and the years of description for those species—we explore whether there are patterns in how newly described species are added to different clades across the history of taxonomic study. In combination, these data allow for a characterization of the impact of past species descriptions on the topology and branch lengths of the clade to which these species belong. Such analyses also allow for informed predictions of how new species might be added to a given clade in the future.

## Materials and methods

### Approach

We utilize recently compiled large species-level phylogenies across the Tree of Life (>2000 species) and the year of scientific description for each species to track when and where species are added to the phylogeny. These data allow us, for example, to determine how the length, shape, and size of phylogenies vary across the history of taxonomic knowledge over the past ∼250 years. We can then ask whether an observed pattern of adding new species between two time-points is different from that expected by adding the same number of species to random positions in the phylogeny. To aid in the evaluation of these patterns, we developed a heuristic approach for assessing this impact of taxonomic practice on characterizing a given phylogeny.

Because taxon sampling clearly impacts our ability to infer the topology and branch lengths in a phylogeny (Heath et al. 2008a, 2008b), a future extension of this approach is to explore how adding newly described species impacts the inference of a phylogeny across different time-points in taxonomic knowledge. Clearly, much of the early history of taxonomic study was conducted without an explicit phylogenetic context and many authors today use phylogenies as an important component in recognizing new species. It is widely acknowledged that phylogenetic relationships change as species are added (Heath et al. 2008a, 2008b) and thus our approach assumes that the most completely sampled tree is the likeliest to represent the “true” relationships. Our analyses do not attempt to track the effect that species addition has on the inference of phylogenetic relationships among species, only the changing shape of this “true” tree as taxonomic sampling grows. Last, because we use metrics developed for characterizing bifurcating phylogenies, we restrict our examples to Eukaryota and do not extend it to Archaea and Bacteria in which reticulation among lineages is likely extremely common (e.g., Gogarten and Townsend 2005).

### Data sources

We focus primarily on two diverse vertebrate assemblages—amphibians and squamate reptiles (lizards and snakes)—because (a) large-scale time-calibrated phylogenies are available for both groups (Pyron and Wiens 2013; Pyron and Burbrink 2014) and (b) these are higher taxa that are currently the focus of systematic revision and species discovery. For example, over the past decade an average of ∼154 new amphibian species was described each year (SD ±23; 2009–2018; AmphibiaWeb 2018), resulting in nearly a 25% increase of the total species diversity over that time. These phylogenies were subsampled to recover circumscribed taxonomic and geographic groups using the R statistical computing environment 3.6.1 (R Core Team 2019). Tree manipulations used functions extract.clade and treedata from ape (v5.3, Paradis and Schliep 2018) and geiger (2.0.6.2, Harmon et al. 2008) packages, respectively. While these are the most complete time-calibrated phylogenies available for these taxa, there are still many described species that are not sampled. For this exercise, we ignore those described species that are unsampled in these large molecular phylogenies. The recently published phylogenies of Jetz and Pyron (2018) and Tonini et al. (2016) were excluded from our analyses because their method of inserting unsampled taxa at the base of clades creates polytomies and obfuscates the effect of taxonomic addition on the shape of the phylogeny.

We utilized the primary resources for taxonomic data on amphibians Frost (2019) and reptiles (Uetz and Hošek 2015) to determine the year of description for each species included in the phylogeny. In addition, we used Uetz and Hošek (2015) to characterize the geographic location of the type locality to provide an example of using these same data to characterize the impact of species discovery in a given geographic region.

To demonstrate the generality of this approach to other animals and plants, we conducted similar analyses for exemplar clades from both flowering plants and invertebrates. For flowering plants, we focus on two large subclades of the Saxifragales—Crassulaceae (stonecrop family) and Saxifragaceae (saxifrage family)—using the phylogeny from Soltis et al. (2013) and taxonomic authorities gathered from the Darwin Core Archive for the Plant List (doi: 10.15468/btkum2). For invertebrates, we combined data on the arachnid order Cyphophthalmi (mite harvestmen) and on Onychophora (velvet worms). Data for Cyphophthalmi were obtained from two sources (Giribet et al. 2012, 2016) and taxonomic authorities collected from the literature by one of us (G.G.); this group was first described in the 18th Century and now has a nearly complete phylogeny and the species diversity has increased by nearly 40% since 2000 (Giribet 2000). For Onychophora, we used a recent time-calibrated phylogeny (Giribet et al. 2018) and a recently updated catalog of species (Oliveira et al. 2012); for the analyses presented here, we removed undescribed species for simplicity. For both Saxifragales and Cyphophthalmi, we converted molecular phylogenies to chonograms using the *chronopl* function in ape (with age.max = 1).

### Metrics

To track the effects of taxonomic addition over the past ∼250 years of taxonomic study, we employed several existing and newly developed functions in R (Supplemental Materials). We pruned the full phylogeny (*T*) to contain only currently recognized species described between 1758 (the year that Linnaeus published the tenth edition of *Systema Naturæ*) and the year that the third species of *T* was described (*n*_1_) using the treedata function in geiger. This returns a “minimum” tree—*T*_1_—with three or more species (although *T*_1_ could contain more than three taxa when multiple species were described in the same year as the third species). We then stored this tree and used a function to calculate several metrics to characterize phylogeny, including the average, maximum, and minimum length of all branches (BL) added that year, the γ-statistic value (γ; Pybus and Harvey 2000), and phylogenetic diversity (PD; Faith 1992), which are further detailed below. We looped this function to record the statistics for each *T_n_* between the years of the first and last species descriptions in *T*. These metrics provide a multifaceted view of how phylogenies change with increasing knowledge of species diversity and quantify different aspects of tree shape (BL and γ). Both R code and data are available via GitHub: https://github.com/drScanley/PhyloDon.

The first metric, new branch-lengths (BL), records the BLs for all newly added species between *T_n_* and *T_n_*_+1_ and returns the mean, maximum, and minimum of the lengths of newly added branches per year.

Pybus and Harvey’s (2000) *γ* is a measure of the distribution of internodes from the root to the tips, with positive or negative *γ* values indicating a concentration of nodes close to the tips or the root, respectively. We calculated *γ* using the ltt function in geiger. Because values for *γ* change as species are added to a phylogeny (Missa et al. 2016), we created a null distribution of *γ* for each *T_n_* by pruning a randomized subset of species from *T* equal to the number of species missing in *T_n_* with 1000 replicates. The looped code records *γ* and both the 2.5% and 97.5% percentiles to record 95% of the null *γ* distribution for each *T_n_*. Because the value of *γ* for a clade is a function of the particular topology and number of tips (Phillimore and Price 2008; Cusimano and Renner 2010), we calculated the observed–expected *γ*, which we call *γ*_OE_, to facilitate comparisons. Two phylogenies with the same topology and number of tips may differ in the observed curve of *γ* because of differing distributions of branch lengths. However, the pattern of *γ*_OE_ remains generally the same (Supplemental Materials). To assess stability in the impact of adding new species to the phylogeny of a clade, we measured the slope of *γ*_OE_ for the past 30 years. A positive slope reveals a recent extended period in which new species are added close to the tips of the tree.

We calculated PD (Faith 1992)—the summed length of the branches of the minimum spanning tree containing only a subset of species (e.g., species added in a given time interval)—to measure the evolutionary breadth of new species added each year. This supplements information from our analyses of how BL changes for a clade across years of taxonomic study by revealing whether new branches tend to be added to small subclades or instead are distributed across the phylogeny, similar to how PD is used in conservation biology studies for a geographic region (Winter et al. 2013). We recorded PD by measuring the summed BLs of a subsampled *T* that only included species missing in the previous *T_n_* (i.e., the species described in year *n*). By adding a step function for each loop, *T_n_* could represent 1 year or a span of years of taxonomic study (Supplemental Materials); for our analyses, we calculated the above metrics for 5-year intervals representing that year (*γ*) or the sum (PD) or average (BL) of that year and the preceding 4 years.

Last, to provide an example of how additions of new species impact inferences of trait evolution, we calculated both evolutionary rate (mean-squared independent contrast; Felsenstein 1985) and phylogenetic signal (*λ*; Pagel 1994) of snout–vent length (SVL)—a commonly used measure of body size—at different time points in taxonomic knowledge of both the Pleurodonta and Gekkota using the data from Feldman et al. (2016).

## Results

**Fig. 1:**
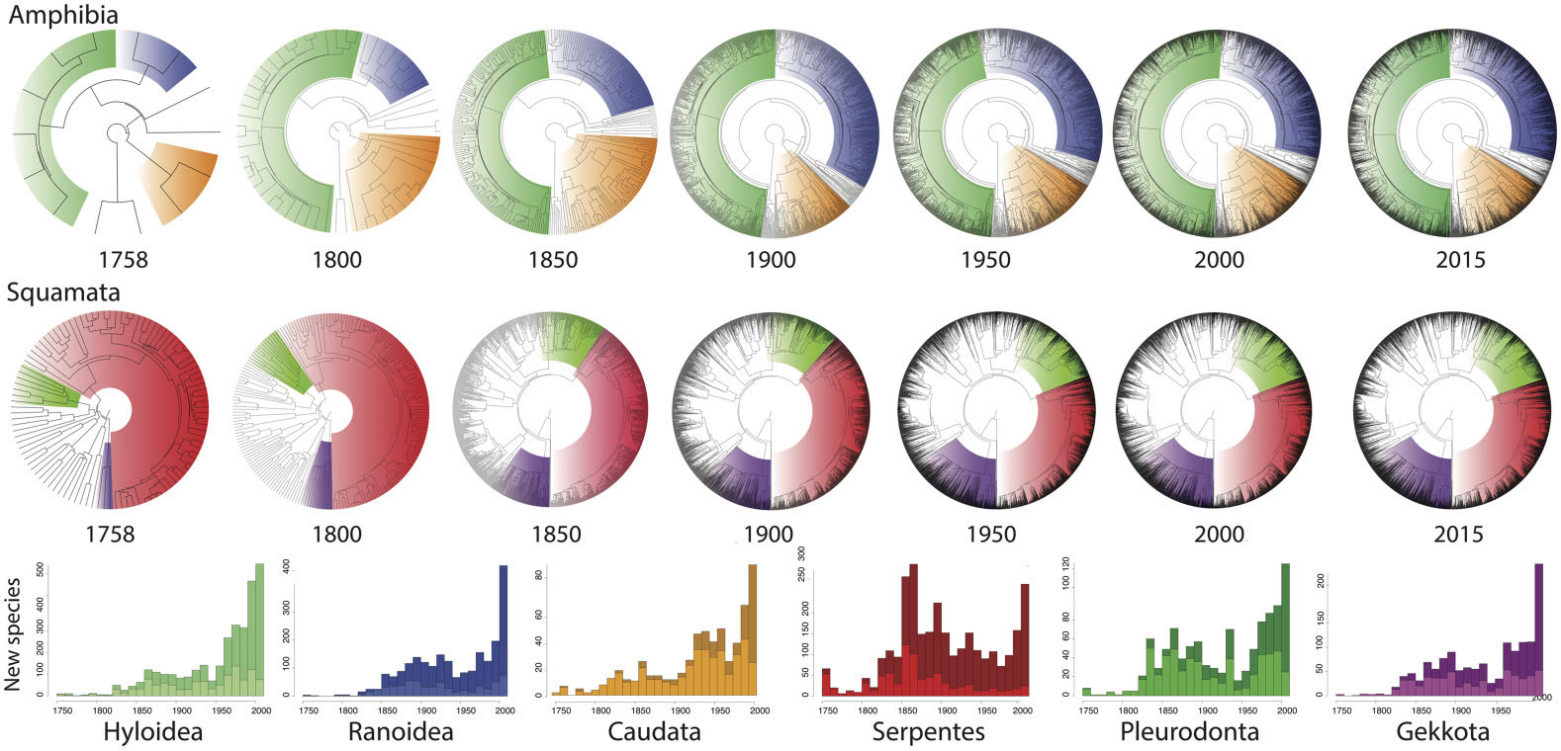
Time-slices in taxonomic knowledge for amphibians (top row) and squamate reptiles (middle row). Bottom row shows comparisons of the number of new species added each year to the phylogeny (light color) in comparison to the total number of new species added each year (dark color) for three clades of amphibians (neobatrachian frogs: Hyloidea [34% of described species], Ranoidea [28%]; all salamanders: Caudata [68%]) and three clades of squamate reptiles (all snakes: Serpentes [28%]; lizards: Pleurodonta [56%], Gekkota [43%]).

**Fig. 2:**
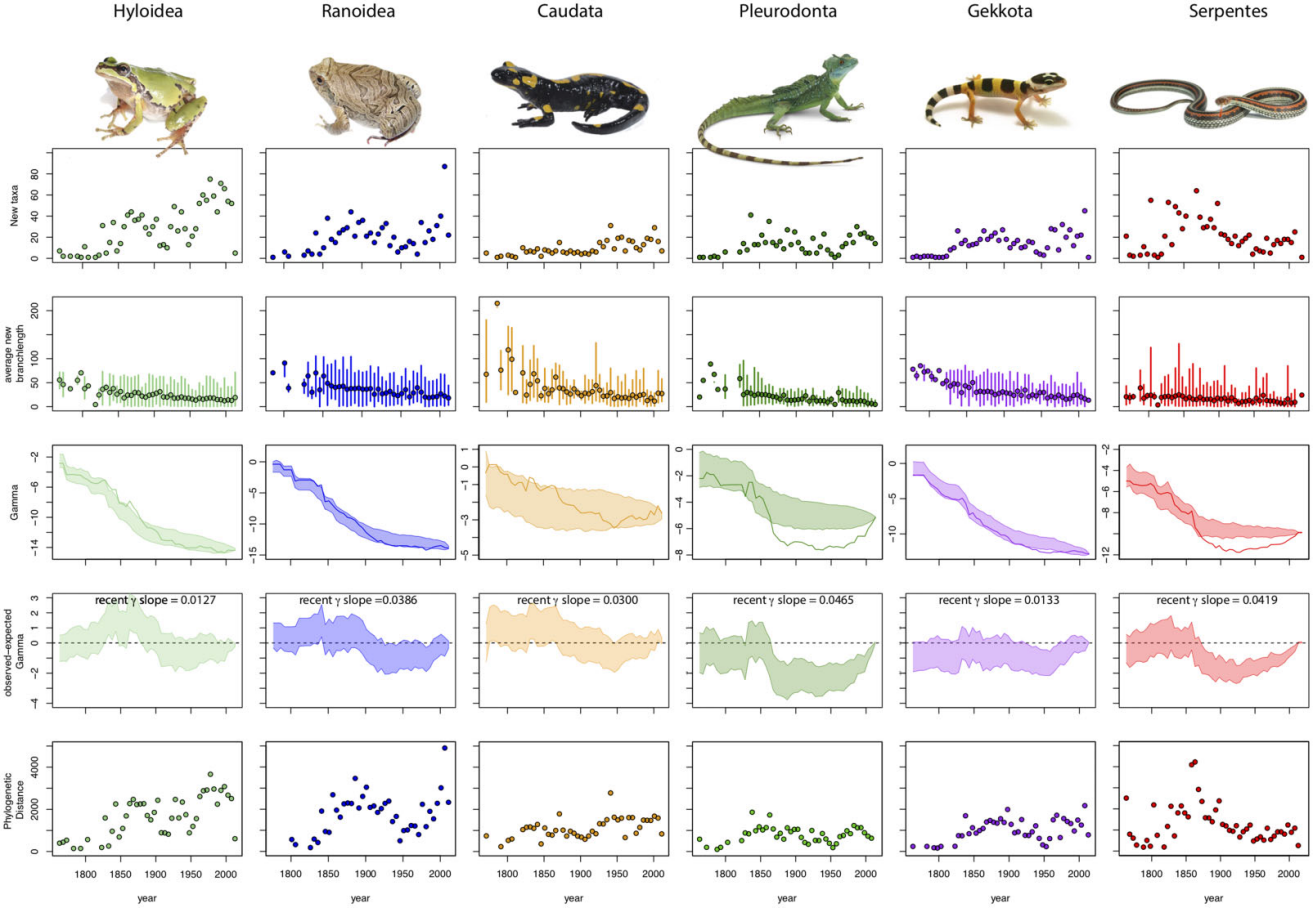
Visualizations of the impact of species discovery on phylogenetic patterns for diverse clades of amphibians and reptiles. Plots for each selected clade show the pattern of discovery of new species in the mean and range of branch lengths (BL), *γ*, observed–expected *γ* (*γ*_OE_), and phylogenetic diversity (PD) across taxonomic history. For each taxon, the plot of *γ* values also states the slope of the *γ*_OE_ for the most recent 30 years. Pleurodonta and Serpentes are well-known clades, whereas new species of Ranoidea and Gekkota continue to have large impacts on major patterns in the phylogeny.

**Fig. 3:**
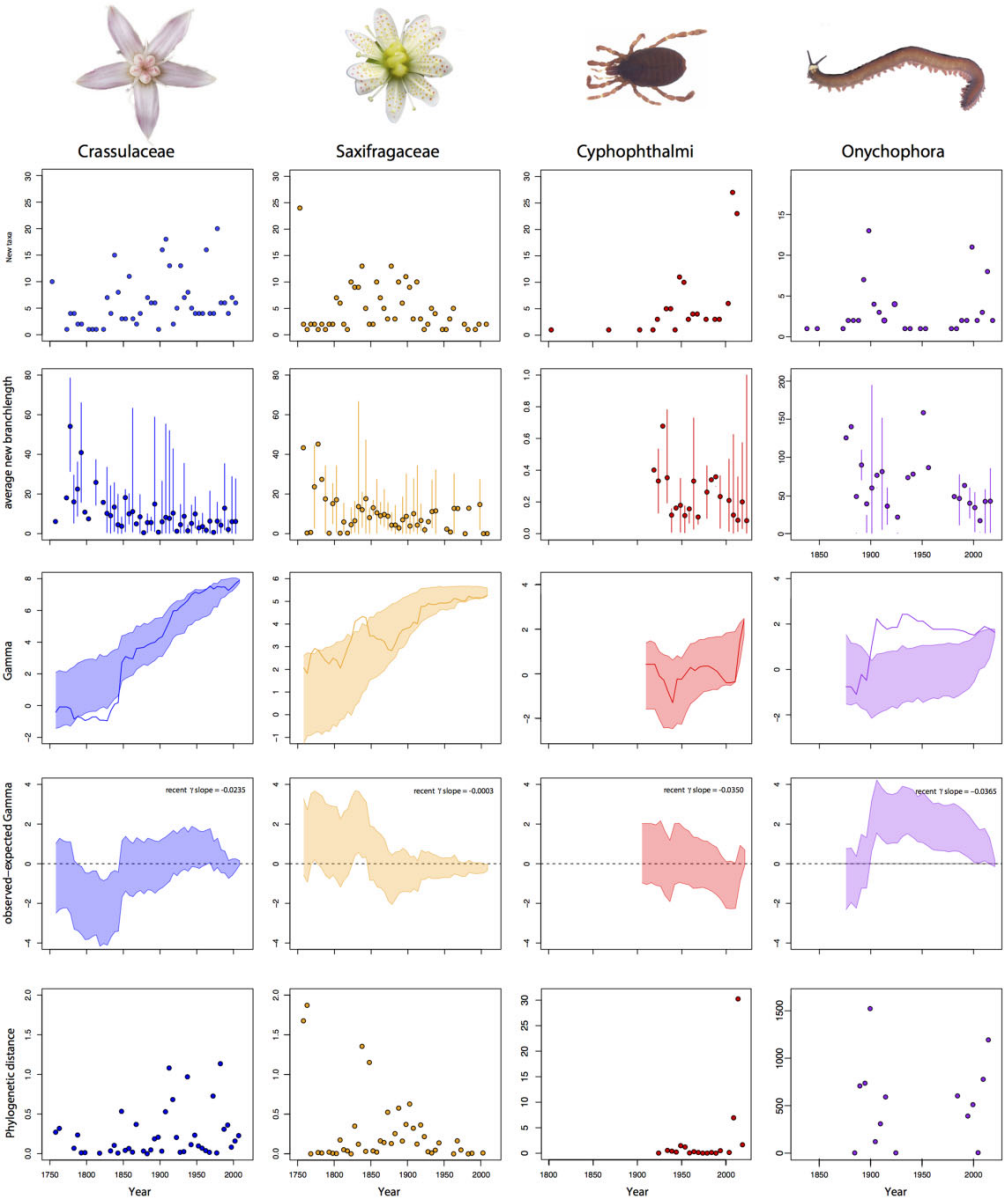
Visualizations of the impact of species discovery on phylogenetic patterns for herbaceous flowering plants and invertebrates. Plots are as in Fig. 2.

**Fig. 4:**
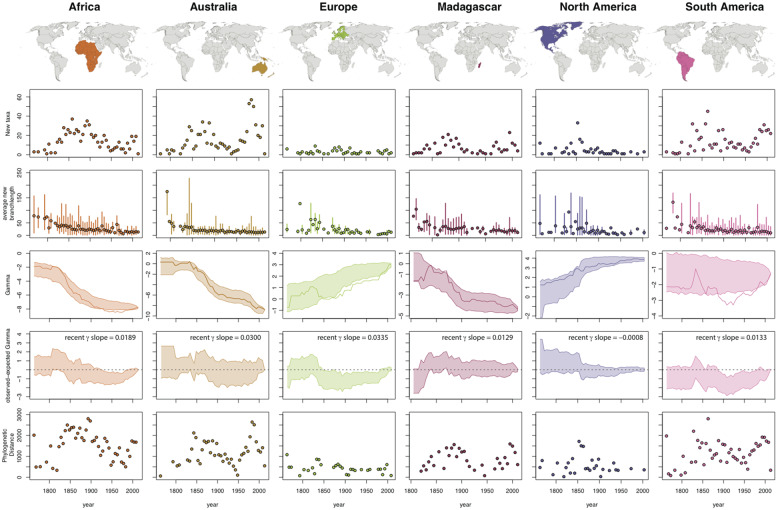
Impact of species discovery on phylogenies of squamate reptiles in three geographic regions. Plots are as in Fig. 2.

We generated phylogenies representing time slices in taxonomic knowledge from Linnaeus to the present based on our current understanding of phylogeny (summarized in Fig. 1). As newly discovered species are added to clades, most show declines in the mean and range of branch lengths (BL) added (Fig. 2). This is intuitive because as a clade becomes better known, newly added species are, on average, more closely related to ones that are already known and thus newly added branch lengths are smaller. However, this pattern varies and for certain clades, such as those with many cryptic species, we observe high variation in BL added (e.g., Ranoidea, Gekkota, Cyphophthalmi; Figs. 2, 3), including across recent years. In addition, the patterns observed for different geographic regions (Fig. 4) make intuitive sense because well-studied temperate regions such as North America and Europe exhibit low values of both the mean and range of BL during the 20th century, whereas tropical regions such as South America exhibit substantial variation in BL across years of taxonomic study up to the present.

Estimates of *γ* vary across different time-points in taxonomic knowledge. Although the overall pattern of *γ* is dependent on tree shape and number of species, the temporal pattern of *γ* appears consistent across most clades examined here. In most taxonomic and geographic groups, we found that *γ* values become increasingly negative as more species are added to the phylogeny (indicating a greater density of nodes near the root), although this is not the case for some clades or regions (e.g., Europe, South America). By generating plots of *γ*_OE_, we can identify periods when the observed *γ* falls outside of the range of *γ* when an equivalent number of random taxa are removed from the original tree. Although tree shape (as measured by *γ*) does not differ significantly over taxonomic history from the null expectation in several large clades, many clades display a clear pattern in which new species are added to the phylogeny. For example, moving forward through time from the mid-18th century to the present day, the observed *γ* value of a number of clades (e.g., Pleurodonta, Serpentes, Crassulaceae) and regional assemblages (e.g., South America) becomes significantly more negative than expected, followed by a period of stasis where the difference between observed and expected *γ* (*γ*_OE_) remains relatively constant, and then increases in the recent past (Figs. 2–4). This result generally reflects the intense effort of describing major lineages during the second half of the 19th century, as well as a late-20th century tendency to describe cryptic species using non-morphological (e.g., DNA sequence) data.

A recent positive slope of *γ*_OE_ (Fig. 2) indicates that recently described species are added close to the tips of the tree. This is supported by comparison to plots of BL showing that well known clades with recent positive slopes of *γ* also have declined in values of BL across recent years.

Variation in PD values across years (Figs. 2, 3) reveals the extent to which taxonomic effort in a given year is distributed across a phylogeny. For some well-known clades, values for PD in recent years reveal a limited phylogenetic scope to the new species described, whereas in other clades new species are being described from a more diverse assemblage of lineages.

**Fig. 5:**
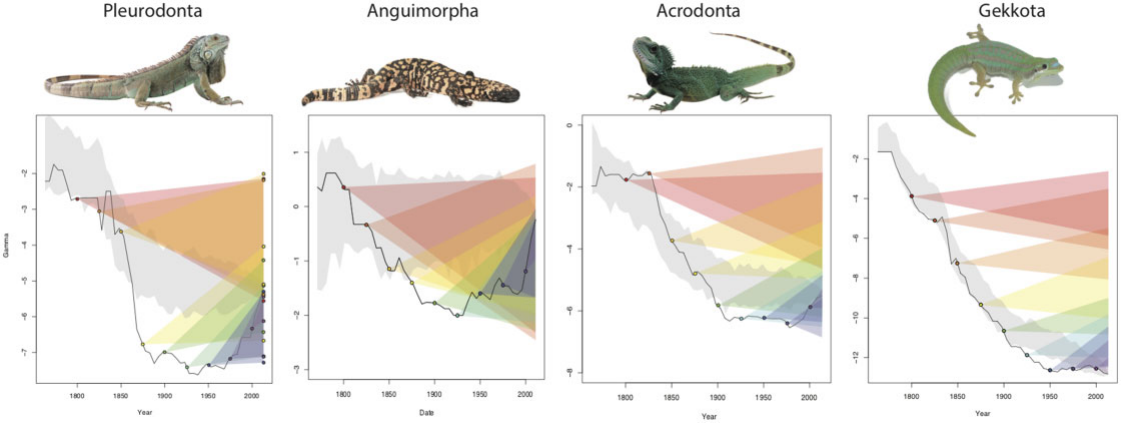
Predicting *γ* values at previous time points in taxonomic knowledge in 50-year intervals. At each time point, the number of missing species from the present-day tree were added 100 times and *γ* calculated on each to create a distribution, which is represented by the different colored intervals mapped at the right of the plot.

When a single continuous character (SVL) is mapped onto the phylogenies of gekkotan and pleurodont lizards, the evolutionary rates and phylogenetic signal vary considerably across taxonomic history (Fig. 5). The estimated evolutionary rate of pleurodont SVL is relatively consistent following the mid 19th century, whereas the estimated evolutionary rate of gekkotan SVL varies three to four-fold over the past 50 years. While estimates of phylogenetic signal, *λ*, would have been largely similar at previous points in taxonomic knowledge for pleurodont SVL, estimates of *λ* for gekkotan SVL ranges from 0 to 1 across this same period.

## Discussion

There is a largely consistent pattern to the impact of adding newly described species to a phylogeny. This pattern of “taxonomic maturation” typically begins with new species being added at random with respect to the phylogeny. Because the early taxonomic history in the clades used here predates phylogenetic and evolutionary approaches to systematics, this pattern might be expected. Following this, some clades exhibit a pattern in which major phylogenetic lineages are described, often with newly described species representing new phylogenetically distinct lineages (e.g., genera or families in our examples). This is reflected in declining values of *γ* (Fig. 2), often dipping below the 95% CI as taxonomists tend to describe species that are more phylogenetically divergent from one another than expected by chance. With major lineages sketched out, addition of new species may proceed largely again at random with respect to the phylogeny. Later in the taxonomic maturation, many clades exhibit a pattern in which newly added species tend to be closely related to already described species. For well-known clades or geographic regions, observed values of *γ* begin increasing in recent years (Figs. 2–4). This reflects a shift from describing species representing long branches to those closely related to other known species. As systematists use a rapidly increasing and powerful toolkit to discriminate among similar species, including data from genes and genomes, mating calls, karyotypes, parasites, and more, we expect that the values for *γ* will continue to increase late in taxonomic maturation. However, this pattern might also be driven by a recent tendency to split species with multiple genetically distinct populations into two or more species (Hillis 2019).

This pattern of taxonomic maturation is most clearly exemplified in our datasets by Pleurodonta, a clade containing nearly 1200 species of conspicuous, largely diurnal, and morphologically diverse lizards that are mostly restricted to the Americas. During 1825–1875, 36 (78%) of the currently recognized pleurodont genera were described, and this is reflected in both the wide range of BL added during this period and the highest PD values during the taxonomic history of pleurodonts (Fig. 2). This group displays a major decline in values of both *γ* and *γ*_OE_ in the second half of the 19th century. This represents the discovery and description of the major pleurodont lineages that accompanied biological exploration in the American Southwest and inland areas of South America, with many currently recognized pleurodont species being described from 1850 to 1900 by systematists such as Spencer F. Baird (*n *=* *15), Marie-Firmin Bocourt (*n *=* *17), George A. Boulenger (*n *=* *37), and Edward D. Cope (*n *=* *80). This was followed by a long period of stasis, in which a range of phylogenetically distinctive taxa were described, and then followed by a period during which molecular tools and other approaches facilitated descriptions of closely related taxa from 2000 to 2019 by systematists such as Cristian S. Abdala (*n *=* *43), Jörn Köhler (*n *=* *54), Andrés S. Quinteros (*n *=* *20), and Jack Sites (*n *=* *20). In the past two decades, PD values for pleurodonts have been relatively low (Fig. 2) largely due to most taxonomic effort being focused within two genera, *Anolis* and *Liolaemus*.

The pattern for Pleurodonta contrasts sharply with Gekkota, a clade of comparable size (nearly 1800 species), but with a worldwide distribution and comprised of often morphologically similar species that are typically nocturnal, making them ecologically cryptic. Gekkota does not display the pattern of taxonomic maturation observed for pleurodonts and the phylogenetic distinctiveness of newly described gekkotan species does not differ from random. This is evidenced by the fact that relatively long branches are still being added across the gekkotan tree, resulting in a wider range of BL and higher values of PD relative to pleurodonts. During the past 50 years, 31 of the 124 currently recognized gekkotan genera were described, including the first species representing nine of these new genera (*Dierogekko*, *Kolekanos*, *Oedodera*, *Orraya*, *Paniegekko*, *Parsigecko*, *Ramigekko*, *Toropuku*, *Tukutuku*). In contrast, only 1 of the 46 currently recognized pleurodont genera was described (*Eurolophosaurus*) during the past 50 years, with its first described species from 1981 (Uetz and Hošek 2015). Because our approach reveals analytically what a gekkotan or pleurodont systematist might tell you based on expert knowledge, this provides a tool for those that are not taxonomically focused experts to obtain a more informed view of a clade before proceeding with analyses that depend on taxon sampling.

**Fig. 6:**
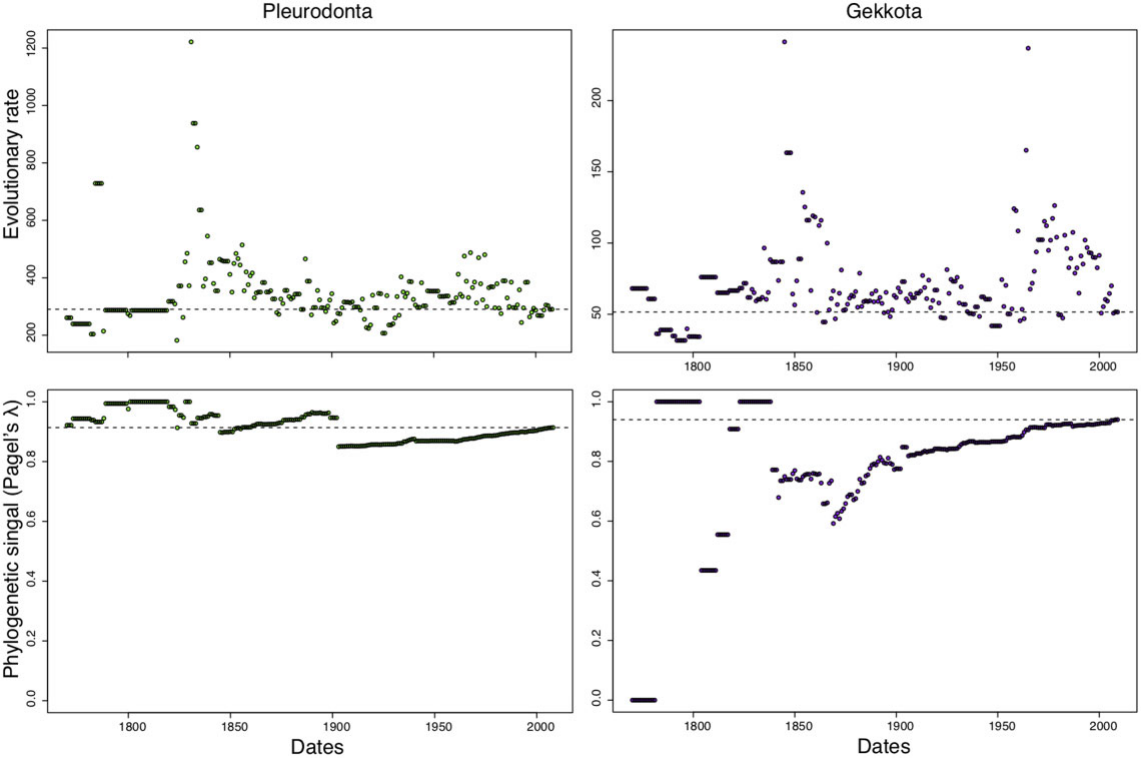
Evolutionary rate (mean-squared phylogenetic independent contrasts) and phylogenetic signal (Pagel’s *λ*) of snout–vent lengths of pleurodont (green) and gekkotan (purple) lizards throughout the taxonomic history of both clades.

Our ability to approximate the “true” tree shape at previous points in taxonomic knowledge depends in part on the pattern of taxonomic maturation. For clades in which early taxonomic descriptions favored species representing deep lineages, we might be able to make predictions for *γ* that encompass the present-day observed value. Using the add.random command in the R package phytools (Revell 2012), we can test how well randomly adding taxa to a phylogeny from a previous time point in taxonomic knowledge recovers the actual present-day *γ*. Such predictions of present-day *γ* do not invariably encompass the observed value (Fig. 6), especially when major lineages remain largely undescribed (e.g., Acrodonta, Gekkota). However, once major lineages are known, especially in well-known clades (e.g., Pleurodonta, Anguimorpha), we can encompass the present-day value for *γ* based on randomly adding tips to phylogenies that represent previous time points in knowledge (Fig. 6). Our ability to accurately predict future values for *γ* then likely depends on the extent to which deep lineages within a clade are known.

The shapes of the *γ* curves observed here (Figs. 2, 3) are similar to those observed in simulated phylogenies by adding species using various models of diversification (Missa et al. 2016). In our analyses, a positive slope of the *γ*_OE_ curve in recent years indicates a period when, on average, new species are being added close to the tips of the phylogeny (and thus values of BL are small). This produces a pattern similar to simulated phylogenies because speciation is happening only at the tips of the trees.

Many scientists focused on diverse clades are faced with the decision of how best to partition their time between describing new species and inferring evolutionary patterns. Is it worth focusing on species discovery and description for a given clade? Or, instead, is the diversity sufficiently known where investigating evolutionary patterns will be worthwhile because most major lineages are probably now known? For well-known clades, we are adding species on average toward the tips of the phylogeny because discovery and addition of major lineages has slowed, but this is not true across the entire Tree of Life. For many clades we are still in an age of major discovery where we will likely add deep branches to the phylogeny (e.g., the cryptic invertebrate lineages used here, Cyphophthalami and Onychophora; Fig. 3). As demonstrated here, we can predict clades or geographic regions that might be understudied or for which new taxonomic efforts might most dramatically alter our understanding of the phylogeny.

Our approach can be used to characterize the extent to which the major lineages of a phylogeny are known as well as predict clades and regions for which future taxonomic effort might have high impact on evolutionary studies. If the variation in branch lengths across recent years remains high and the slope of observed *γ* over the past decade is similar to the null, then authors should evaluate the robustness of evolutionary inferences for a clade by randomly adding tips representing potential new species across the phylogeny. In contrast, if branch length variation across recent years is small and the slope of *γ*_OE_ is positive over the recent past, then it might be more realistic to represent unsampled and undescribed species by concentrating them toward the tips of the tree when simulating their addition to the phylogeny. When selecting clades on which to conduct analyses of lineage or trait diversification (Harmon et al. 2010), our approach can be used to determine those clades for which evolutionary inferences might be more robust to future species descriptions.

The proportion of taxonomic “known unknowns” is a definable value that is constantly eroded as described species are placed in a phylogenetic framework. Yet it is difficult to predict the proportion and placement of missing “unknown unknowns” (Pimm 2012) and the effect that these missing taxa might have on evolutionary analyses (Figs. 2, 3, 6). Even for described taxa, variation in taxonomic opinion among authors might affect the results of diversification analyses (Faurby et al. 2016), and the ways in which authors accommodate “unknown unknowns” could have equally major impacts on their evolutionary inferences. By incorporating an understanding of taxonomic maturation into analyses, we will build on previous work characterizing biases in species descriptions (Roberts and Marshall 2009). We recommend that authors evaluate whether evolutionary inferences (e.g., *γ*, *λ*) differ based on adding “unknown unknowns” to their focal phylogeny informed by whether most newly described species are added close to the tips or instead are more evenly distributed across the depth of the tree.

## Supplementary Material

obz028_Supplementary_Data
