# Corrigendum

**DOI:** 10.1093/iob/obz034

**Published:** 2019-12-28

Corrigendum to: Predicting the Impact of Describing New Species on Phylogenetic Patterns by D. C. Blackburn, G. Giribet, D. E. Soltis and E. L. Stanley

In “Predicting the Impact of Describing New Species on Phylogenetic Patterns” [*Integrative Organismal Biology*, Volume 1, Issue 1, 2019, obz028, https://doi.org/10.1093/iob/obz028], the legends for Figures 5 and 6 were inadvertently swapped. These have now been corrected in the article.

