# Supplementary material for: Predicting the Impact of Describing New Species on Phylogenetic Patterns
Source: Integr Org Biol. 2019 Nov 7;1(1):obz028. doi: 10.1093/iob/obz028 (PMC7671110; doi:10.1093/iob/obz028)
Supplement: obz028_Supplementary_Data [file obz028_supplementary_data.zip › Supp-mat 1 Gamma adjust.pdf]

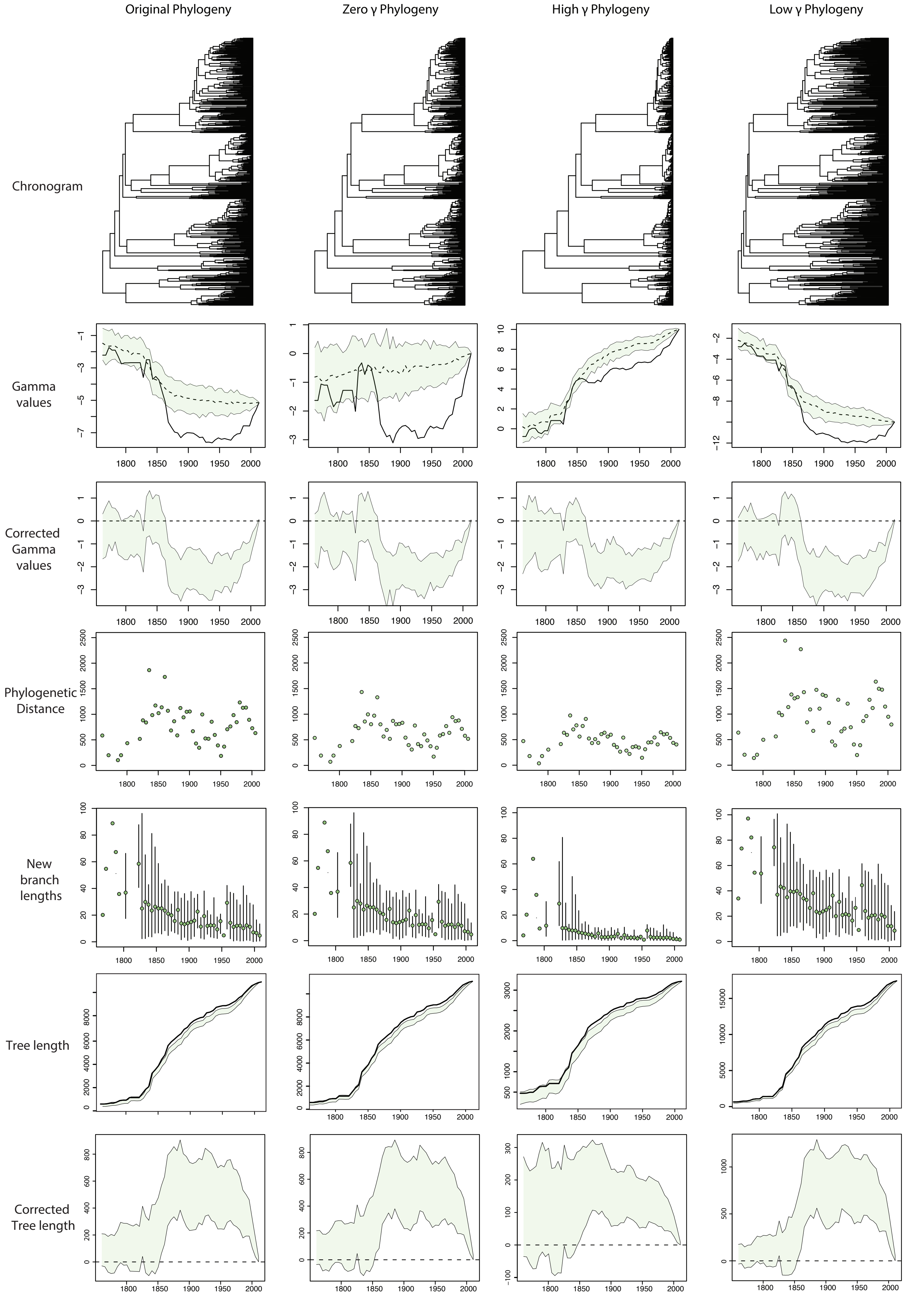

Original Phylogeny
Zero γ Phylogeny
High γ Phylogeny
Low γ Phylogeny
Chronogram
Gamma values
Corrected Gamma values
Phylogenetic Distance
New branch lengths
Tree length
Corrected Tree length
