# Supplementary material for: Predicting the Impact of Describing New Species on Phylogenetic Patterns
Source: Integr Org Biol. 2019 Nov 7;1(1):obz028. doi: 10.1093/iob/obz028 (PMC7671110; doi:10.1093/iob/obz028)
Supplement: obz028_Supplementary_Data [file obz028_supplementary_data.zip › suppmat_v3.docx]

**Supplemental Materials**

While two trees with the same topology and number of tips might differ in values of γ based on the distribution of branch lengths across the tree, the ratio between the observed and the expected gamma should remain consistent. To exemplify this, we have chosen one phylogeny (for iguanian lizards) and transformed the original tree to have, respectively, high, low, and zero value for gamma. Whereas the plot of observed gamma values changes through time, the plot of observed-minus-expected-gamma is nearly identical for each (Supplemental Figure 1). While the shape of the phylogeny affects values of gamma across the time will change between can differ among phylogenies with the same number of tips and topology, plots of observed–expected γ remain largely similar and provide a useful tool for comparing among phylogenies.

Trees were constrained to specific γ values GammaModify, a function derived from code generously provided by Liam Revell.

—

GammaModify<-function(tree,r=c(-10,10),g=0)

{ ebTree<-function(tree,r){

if(r!=0){

H<-nodeHeights(tree)

e<-(exp(r*H[,2])-exp(r*H[,1]))/r

tree$edge.length<-e

}

tree

}

EB<-function(tree,r){

d<-max(nodeHeights(tree))

tree<-ebTree(tree,r)

tree$edge.length<-tree$edge.length/max(nodeHeights(tree))*d

tree

}

gamma<-function(r,tree,g)

{(g-ltt(EB(tree,r),plot=FALSE)$gamma)^2}

fit<-optimize(gamma,c(-10,10),tree=tree,g=g,tol=1e-12)

EB(tree,fit$minimum)->ModifiedGTree

return(ModifiedGTree)

}

**Supplemental Figure 1.**

Analysis performed on A) phylogeny for Pleurodonta, and the same tree with modified branch-lengths to produce trees with specific Pybus and Harvey’s γ: B) γ = 0, C) γ = 10, D) γ = -10. The second row shows the γ values of actual taxonomic subtractions (solid line) and 95% of γ values for the same phylogeny with randomized taxa subtracted at the same rate (green polygon) for the various trees. The third row shows this randomized subtraction γ minus the observed γ. In all scenarios, the γ value trends towards zero as the taxa are removed, leading to different looking plots in the second row. However, the relationship between the observed-minus-expected-gamma remains highly consistent regardless of the final γ value, suggesting that the signal of taxonomic prioritization is not influenced by treeshape. The fourth and fifth rows show the phylogenetic distance and new branch length values, respectively, for the four trees. Again, modifying the γ of the final trees modify the values but not the overall pattern of these metrics. The fifth row shows the cumulative observed treelength (solid line) and the 95% range of treelength from 1000 randomized trees (the expected range if species description rates were random). The sixth row shows the relationship between the observed and expected treelength, which exhibit the same temporal patterns as the observed/expected γ plots. This is unsurprising as both of these metrics are influenced by branch length distributions: preferentially describing phylogenetically distinct taxa, as seen in from 1850 to1900 on these plots, lowers γ and increases treelength rapidly, while taxonomic splitting at the tips of the tree, as seen from 1970 onwards, raises γ and increases the treelength less quickly.

**Supplemental Figure 2.**

In our analyses, we used a bin size of 5 years for the interval over which we calculated Phylogenetic Diversity (PD). This was largely done for heuristic purposes, though users of our R code could generate bins of different sizes to explore how this influences plots of PD over time. However, as shown in this example for Pleurodonta, the general patterns of PD are similar through time regardless of the number of years (1–10) in the binning intervals shown here.

**Supplemental Figure 3.**

In order to demonstrate that the signal of early addition of deep lineages and late addition of shallow lineages seen in the Iguanid tree is derived from patterns in the data and not simply random chance, we compared the results from the original Gamma analyses with a dataset of randomized description dates on the tree tips. As expected, the observed gamma values for the perturbed dataset do not differ from random, never falling outside of the 95% quantile of the expected gamma range.
