# Supplementary figures and images for: Predicting the Impact of Describing New Species on Phylogenetic Patterns
Source: Integr Org Biol. 2019 Nov 7;1(1):obz028. doi: 10.1093/iob/obz028 (PMC7671110; doi:10.1093/iob/obz028)

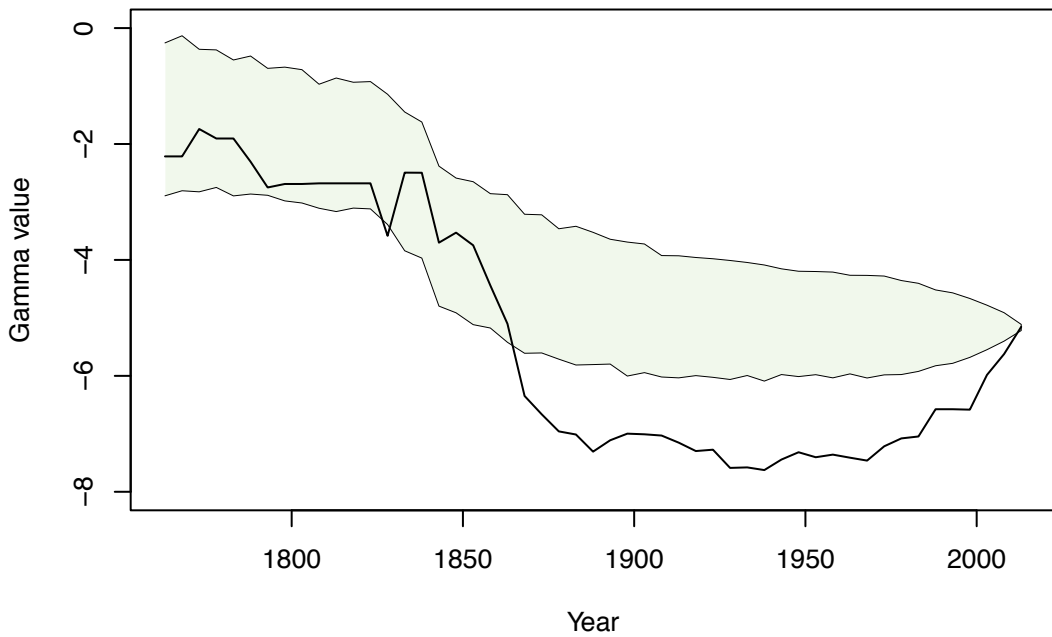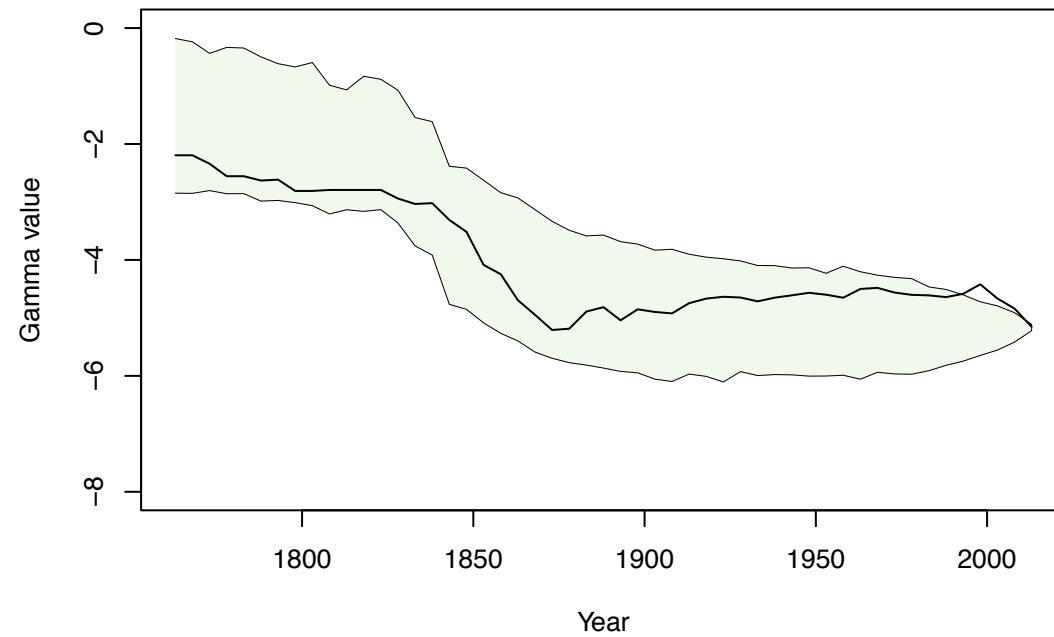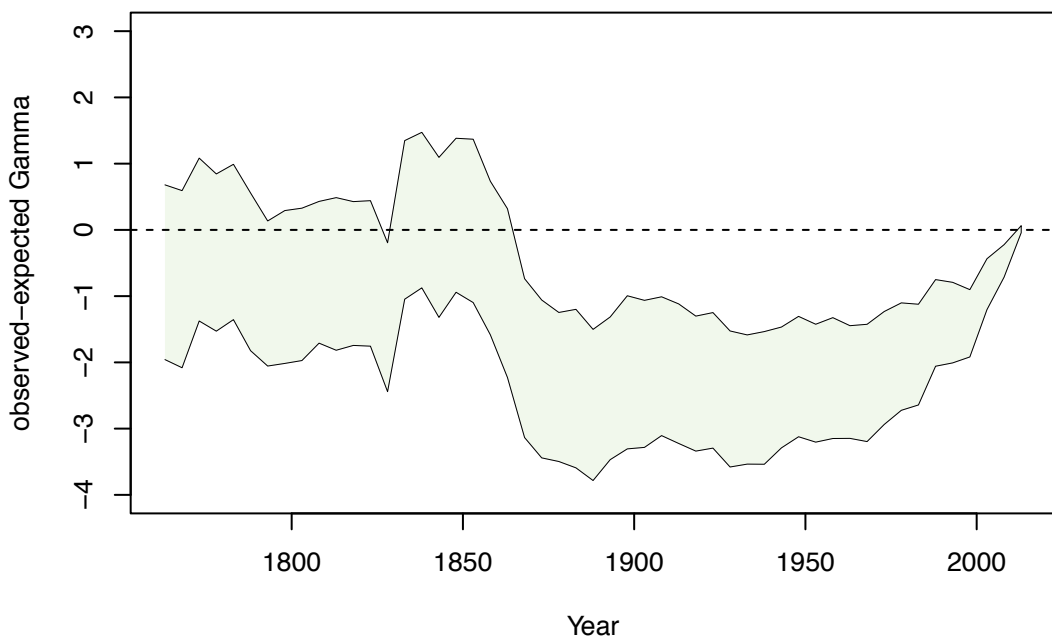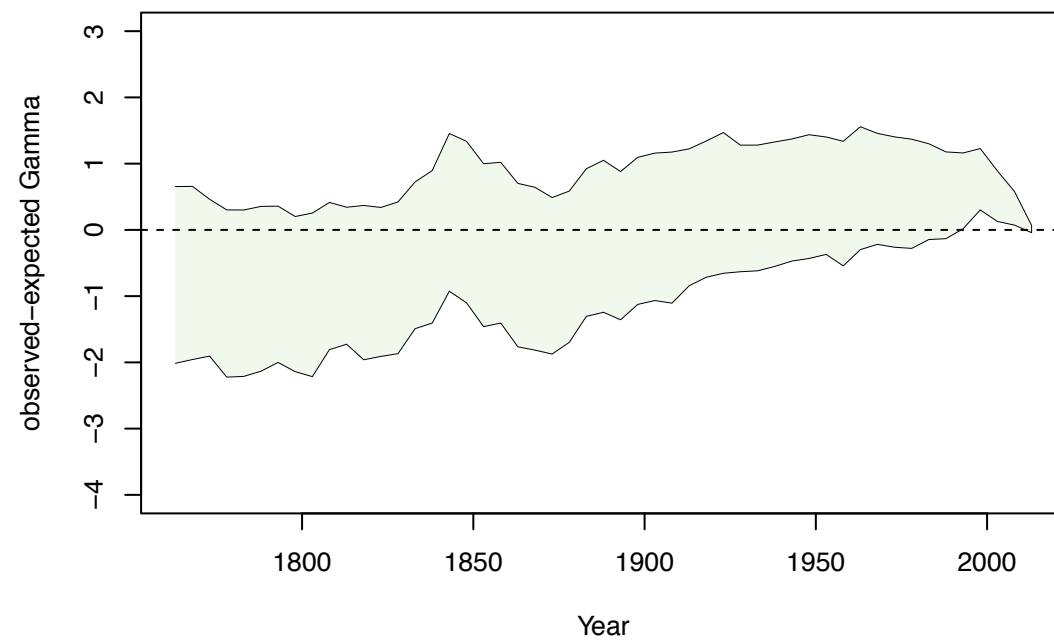

Supplement: obz028_Supplementary_Data [file obz028_supplementary_data.zip › Supp-mat 3 Randomized Dates.pdf]

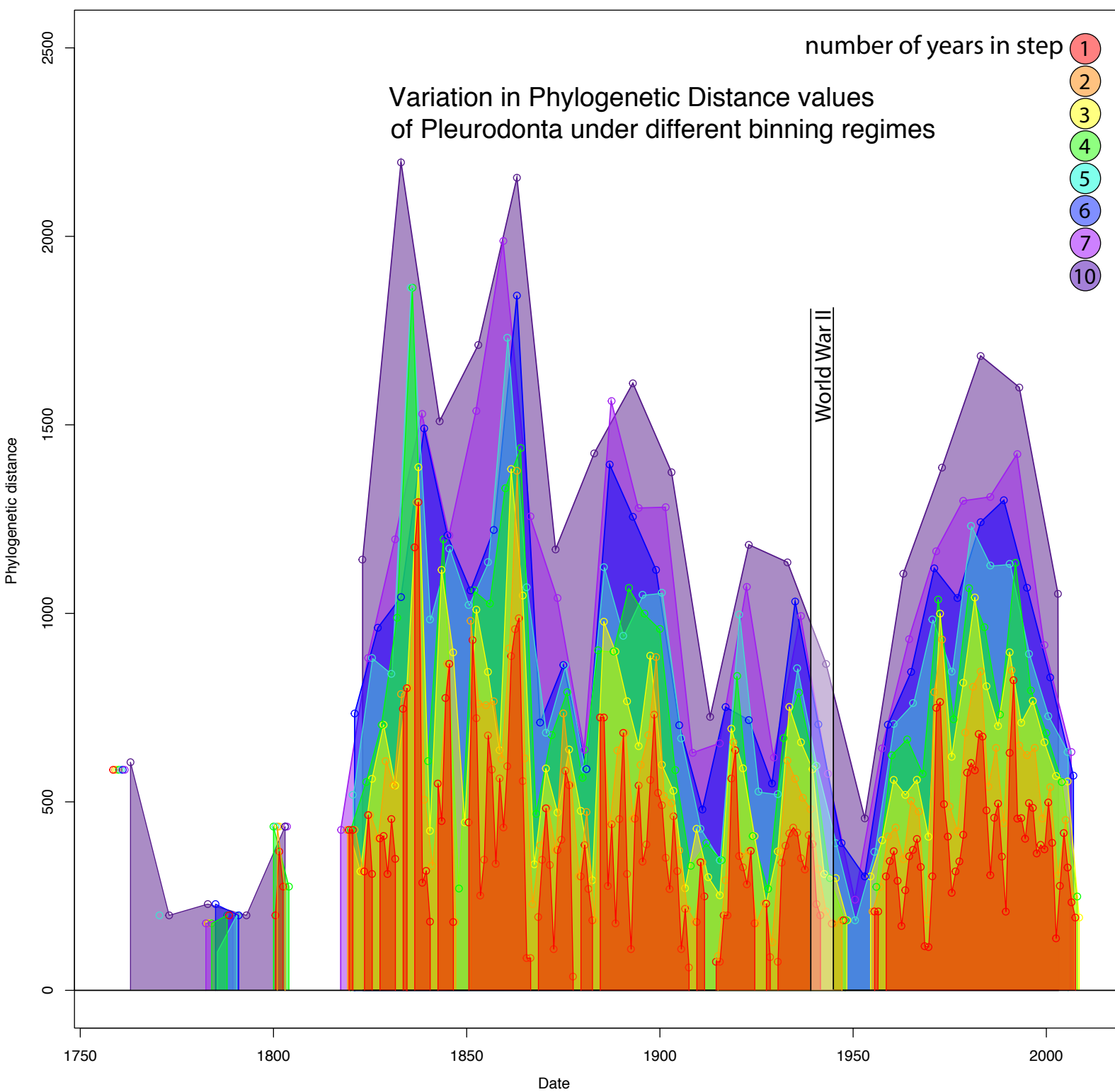

Supplement: obz028_Supplementary_Data [file obz028_supplementary_data.zip › Supp-mat_2_PD_year_window_v2.pdf]
